# Local and systemic defense response in Aspen clones: contrasting defense response to biotrophic and necrotrophic pathogens

**DOI:** 10.1186/1753-6561-5-S7-P83

**Published:** 2011-09-13

**Authors:** Carl Gunnar Fossdal, Nadeem Yaqoob, Halvor Solheim, Jan Karlsson, Benedicte Riber Albrectsen

**Affiliations:** 1 The Norwegian Forest and Landscape Institute, Norway; 2 Umeå Plant Science Centre, Sweden

## 

Trees are exposed to a variety of pathogenic fungi. The defense response toward a biotroph may require a different strategy that toward a necrotroph. To understand the key processes of defense responses toward pathogenic fungi in aspen (*Populus tremulae*) at the transcript level we inoculated clones of this species with a foliar rust on the leaves and a necrotroph in the bark. Leaf samples were collected from above the inoculation site to examine the long distance (systemic) defense responses and bark tissue around the site of inoculation examined for the local response as early as day1 post treatments. We performed microarray experiments on the biotrophic and necrotrophic interaction and between healthy controls of two SwAsp clones. Selected candidate genes were also examined in more detail by qRT-PCR and chemical analysis for phenols and tannins was also performed. We found that the two clones respond in a very different in fashion at the transcriptional level to both the biotrophic and necrotrophic pathogen. The more resistant clone responded systemically within 24 hours while little response at the transcriptional level was detected in the more susceptible clone in response to the biotroph, while indications of suppression in response to the necrotroph was found.

